# Movement-Related Theta Rhythm in Humans: Coordinating Self-Directed Hippocampal Learning

**DOI:** 10.1371/journal.pbio.1001267

**Published:** 2012-02-28

**Authors:** Raphael Kaplan, Christian F. Doeller, Gareth R. Barnes, Vladimir Litvak, Emrah Düzel, Peter A. Bandettini, Neil Burgess

**Affiliations:** 1 NIMH-UCL Joint Graduate Partnership Program in Neuroscience, Bethesda, Maryland, United States of America; 2 UCL Institute of Cognitive Neuroscience, University College London, London, United Kingdom; 3 Section on Functional Imaging Methods, Laboratory of Brain and Cognition, National Institute of Mental Health, Bethesda, Maryland, United States of America; 4 UCL Institute of Neurology, University College London, London, United Kingdom; 5 Donders Institute for Brain, Cognition and Behaviour, Radboud University Nijmegen, Nijmegen, The Netherlands; 6 Wellcome Trust Centre for Neuroimaging, University College London, London, United Kingdom; 7 Institute of Cognitive Neurology and Dementia Research, Otto von Guericke University, Magdeburg, Germany; 8 German Center for Neurodegenerative Diseases (DZNE), Magdeberg, Germany; Boston University, United States of America

## Abstract

The hippocampus is crucial for episodic or declarative memory and the theta rhythm has been implicated in mnemonic processing, but the functional contribution of theta to memory remains the subject of intense speculation. Recent evidence suggests that the hippocampus might function as a network hub for volitional learning. In contrast to human experiments, electrophysiological recordings in the hippocampus of behaving rodents are dominated by theta oscillations reflecting volitional movement, which has been linked to spatial exploration and encoding. This literature makes the surprising cross-species prediction that the human hippocampal theta rhythm supports memory by coordinating exploratory movements in the service of self-directed learning. We examined the links between theta, spatial exploration, and memory encoding by designing an interactive human spatial navigation paradigm combined with multimodal neuroimaging. We used both non-invasive whole-head Magnetoencephalography (MEG) to look at theta oscillations and Functional Magnetic Resonance Imaging (fMRI) to look at brain regions associated with volitional movement and learning. We found that theta power increases during the self-initiation of virtual movement, additionally correlating with subsequent memory performance and environmental familiarity. Performance-related hippocampal theta increases were observed during a static pre-navigation retrieval phase, where planning for subsequent navigation occurred. Furthermore, periods of the task showing movement-related theta increases showed decreased fMRI activity in the parahippocampus and increased activity in the hippocampus and other brain regions that strikingly overlap with the previously observed volitional learning network (the reverse pattern was seen for stationary periods). These fMRI changes also correlated with participant's performance. Our findings suggest that the human hippocampal theta rhythm supports memory by coordinating exploratory movements in the service of self-directed learning. These findings directly extend the role of the hippocampus in spatial exploration in rodents to human memory and self-directed learning.

## Abstract 2

A multimodal neuroimaging study of virtual spatial navigation extends the role of the hippocampal theta rhythm to human memory and self-directed learning.

## Author Summary

Neural activity both within and across brain regions can oscillate in different frequency ranges (such as alpha, gamma, and theta frequencies), and these different ranges are associated with distinct functions. In behaving rodents, for example, theta rhythms (4–12 Hz) in the hippocampus are prominent during the initiation of movement and have been linked to spatial exploration. Recent evidence in humans, however, suggests that the human hippocampus is involved in guiding self-directed learning. This suggests that the human hippocampal theta rhythm supports memory by coordinating exploratory movements in the service of self-directed learning. In this study, we tested whether there is a human analogue for the movement-initiation-related theta rhythm found in the rodent hippocampus by using a virtual navigation paradigm, combined with non-invasive recordings and functional imaging techniques. Our recordings showed that, indeed, theta power increases are linked to movement initiation. We also examined the relationship to memory encoding, and we found that hippocampal theta oscillations related to pre-retrieval planning predicted memory performance. Imaging results revealed that periods of the task showing movement-related theta also showed increased activity in the hippocampus, as well as other brain regions associated with self-directed learning. These findings directly extend the role of the hippocampal theta rhythm in rodent spatial exploration to human memory and self-directed learning.

## Introduction

Spatial exploration provides an ecologically valid experimental paradigm to investigate volitional behaviour and cognition across different species. In freely behaving rodents, the theta rhythm (∼4–12 Hz) dominates the hippocampal local field potential (LFP) during translational motion, particularly prominent during initiation of movement [1]–[3], and has been associated with the encoding and behavioural control of memories [4]–[5]. Notably, movement-related theta in rodents is modulated by environmental novelty [6] and has shown a correlation between age-related memory decline and decreased amplitude [7]. However, it has been difficult to disambiguate cognitive influences on the rodent hippocampus from effects of movement per se [8]–[9]. In human memory there has been a slightly different examination of volitional behaviour. The ability to self-initiate memory behaviours was observed as a crucial biomarker for age-related memory decline [10] and more recently the human hippocampus was observed to be a network hub for the volitional control of memory encoding [11]. Yet in the electrophysiology domain, human theta research (∼4–8 Hz) has mostly focused on passive declarative or working memory ([12]–[16], reviewed in [17]). Thus the role of theta in self-directed learning and the correspondence between the role of theta in mnemonic processing and in self-initiated movement is unclear. Some studies have measured hippocampal theta during virtual navigation tasks [18]–[21], and these interactive human tasks may allow assessment of the roles of theta in both self-initiated virtual movement and self-directed learning within the same task.

**Figure 1:**
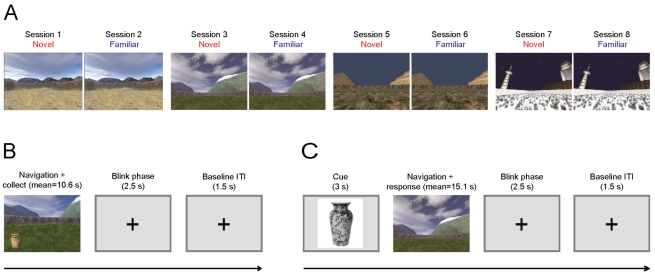
Overview of design and spatial memory task. (A) Experimental environments shown from the participants' (first-person) perspective. Four different environments are presented in eight experimental sessions. The first two sessions (always the desert environments) provided practice outside of the MEG scanner. Sessions 3–8 contained three novel-familiar environment repetitions with environment order, counterbalanced across participants. (B) Learning phase trial structure. During learning trials, participants use a button box to navigate and “collect” novel and familiar (previously presented) objects (vase shown as example). (C) The test phase, trial structure. After being cued for 3 s with a picture of an object that had been collected in the learning phase of the current session, participants were placed back in the environment and had to navigate to where they thought the object had been located during that learning period.

We designed a virtual exploration task that parallels foraging paradigms in rodents and behavioural and fMRI studies in humans [22]–[24]. In our task, participants used a button box to move and explore a total of six novel or familiar environments (like a video game controller, see Figure 1A), while being scanned by a 275 sensor whole-head Magnetoencephalography (MEG) system. During the learning period of an experimental session, participants were instructed to remember (maintain spatial representations of object location) and navigate to the location of an object (either novel or familiar) in a particular trial. At the beginning of each trial, the participant would be placed in different locations within the environment and then use the button box to freely move around the environment. A single trial consists of navigation towards an object and then running over it, which marks the end of that trial (Figure 1B; in each session there were six randomized familiar or novel objects each learned over three trials; see Materials and Methods for details). After completing the learning phase of a session, participants had a test phase for each object's location. In each test trial, participants were cued with a picture of a previously found object from that session's learning phase. Immediately after being cued, participants were placed in the virtual environment and had to navigate to the location where they had encountered the object and press a button (i.e., “replace” it) to conclude the trial (Figure 1C). As a follow-up (on a later date) with the same participants, we used fMRI functional localizer sessions composed of two analogous learning phases with familiar environments and objects. Participants subsequently completed the test phases outside of the fMRI scanner (refer to Materials and Methods for details).

To see whether there is a human analogue for the movement-initiation-related theta rhythm found in the rodent hippocampus (type I theta, refer to [1]), we employed a multi-modal neuroimaging paradigm to look at human memory [25]. We looked for increased MEG theta power and hippocampal fMRI activity during the onset of self-initiated movement at any point within virtual navigation. To better corroborate movement-related theta, we also investigated the effect of environmental novelty with MEG. Additionally, we tested for relationships between subsequent memory performance and theta power, following related findings in humans [13]–[14],[26]–[31] and looked to see how this relationship interacted with any movement-related effects. We were interested to see how areas associated with movement- or performance-related theta effects might overlap with the hippocampal-dependent network observed during the active control of learning [11]. In this way, we aimed to clarify the functional roles of human theta oscillations in cognition and volitional behaviour, and synthesize previous findings in the hippocampus across rodents and humans. Our findings suggest that the theta rhythm supports hippocampal-dependent memory by coordinating exploratory movements in the service of self-directed learning.

## Results

### Behavioural Results

The average lengths of navigation trials in the learning and test periods were 10.6 s and 15.1 s, respectively (averaged over the 20 participants measured). Participants displayed a linear trend of spending less time navigating during learning (*p* = .006, *F*(1, 19) = 9.416) and test (*p* = .015, *F*(1, 19) = 7.105) trials in later experimental sessions. Replacement error (the distance between indicated object location and the correct location within the environment during the test phase) showed a linear trend of decreasing object replacement distance over the whole experiment (i.e., improving performance, *p* = .027, *F*(1, 19) = 5.84), but no significant change in error within any individual session (*p* = .141, *F*(6, 19) = 1.707, see Figure S1). There was no significant difference in replacement error between new and familiar environments (*p* = .141, *t*(19) = −1.535), but performance for novel objects was significantly better than for familiar objects (*p* = .023, *t*(19) = 2.464). The better memory performance for novel objects was not surprising, since familiar objects were used in a different location in a previous environment, which could lead to source interference (see Figure S1).

### Time-Frequency Analyses

#### Movement initiation analyses

**Figure 2:**
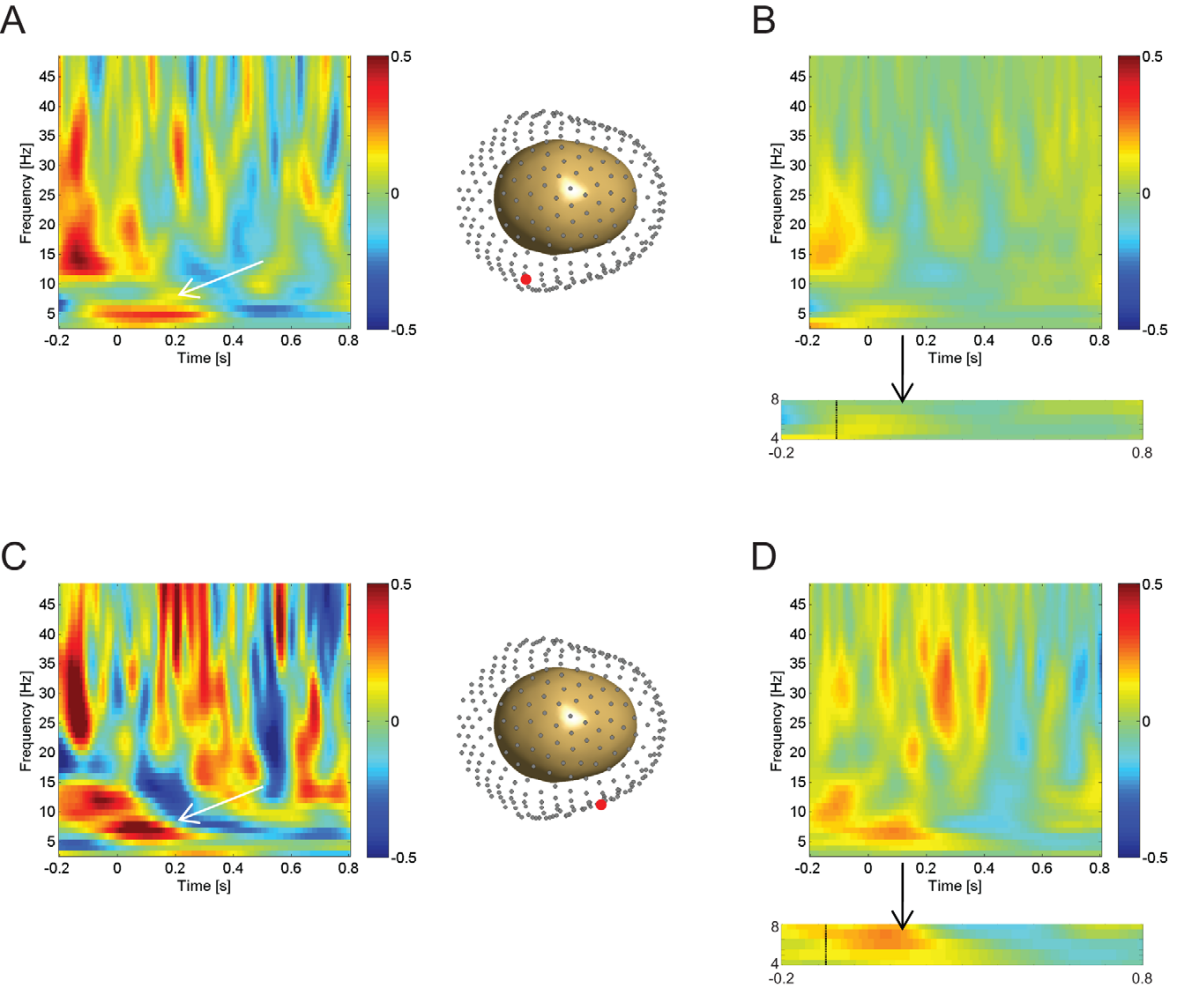
Movement-related MEG time-frequency effects. Plots show MEG signal as baseline corrected log normalized difference scores (dB, z axis) against time (*x*-axis, seconds) and frequency (*y*-axis, Hz), averaged across participants (*n* = 18). (A) The effect of movement initiation during navigation (left panel) at an exemplary single sensor of interest, MRT16, whose posterior right middle temporal location is highlighted in red in a 3-D representation of the MEG sensors around the head (right panel). (B) The effect of movement initiation averaged across all 270 MEG sensors (top). Box below highlights the theta band (4–8 Hz), the vertical line (∼−50 ms) signifies the start of the significant temporal cluster within the theta band (FWE *p*<.05). (C) The effect of environmental familiarity on the movement initiation effect (movement initiation in a familiar environment versus movement initiation in a novel environment) at an exemplary single sensor of interest, MRF56 (left), whose middle right frontal location is highlighted in red (right). (D) The effect of environmental familiarity on the movement initiation effect averaged across all 270 MEG sensors. Box below (format as Figure 2B) shows effect at ∼−83 ms within the theta band (FWE *p*<.05).

In order to perform an event-related characterization of the data, we analysed 1-s periods of movement onset or being stationary within any point of a navigation trial using five-cycle Morlet wavelets (see Figure S2; refer to Materials and Methods for details). We refer to 1-s periods of movement onset (initiation) and stationary periods during navigation as “epochs.” These 1-s time periods were used in MEG analysis during navigation across participants (*n* = 18) for the movement onset analysis. We used paired *t* tests to compare power during different types of epochs over multiple trials, where we looked for theta effects averaged across all sensors. Here and in the novelty analysis below we used a significance threshold of *p*<.05 Family Wise Error (FWE) cluster corrected for multiple comparisons (over time and frequency) for both the movement and novelty effects [32]. There was a significant difference in theta power (i.e., 4–8 Hz power averaged over all sensors) for movement onset compared to stationary epochs, that peaked at ∼−50 ms (cluster-level FWE corrected *p*<.040, *t*(17) = 5.07) before the onset of movement (Figure 2A–B). This demonstrated a significant increase in transient-induced theta power (centered around ∼5 Hz) preceding a 20 Hz (beta frequency) button press-related power increase. In both traces at the sensor level, the oscillatory nature of these differences is apparent (see Figure S3 for an example). There was also activity at 14–16 Hz approximately 200 ms before movement, concurrent with an extension of the theta cluster. The oscillatory and temporal topography of the beta and theta oscillations on the time frequency plot (see Figure 2) parallels previous findings separating movement-related power changes during spatial wayfinding from simple motor planning during navigation experiments [33].

#### Novelty analyses

We also tested the persistence of movement initiation effects by comparing the effect with regards to environmental and object novelty. A 2×2 ANOVA with factors of environmental and object novelty was performed on the time-frequency signal from all sensors for movement onset versus stationary epochs. Increased theta power was found for familiar versus novel environments during the initiation of movement, with the peak increase beginning at −83 ms (cluster-level FWE *p*<.048, *t*(17) = 4.15). However, no theta power increases were observed for the reverse contrast (novel versus familiar environments) or any effects due to the contrast between object novelty versus familiarity (Figure 2C–D). The increased theta power related to movement initiation in familiar versus novel environments resembled that for the movement onset effect alone, but was stronger and covered a wider range of frequencies within the theta band.

### Performance Effects

**Figure 3:**
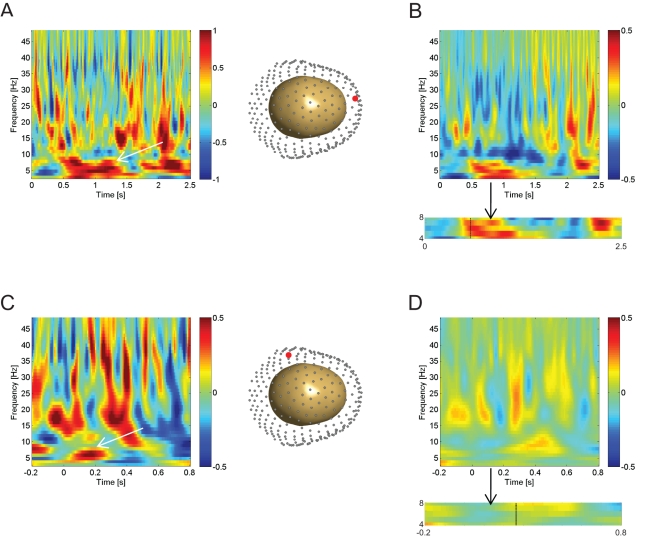
Performance-related MEG time-frequency effects. Subsequent performance effects are shown as the difference between time-frequency spectra for accurate trials versus inaccurate trials. Trials were divided into accurate and inaccurate according to whether replacement accuracy for that trial was above or below the participant's median accuracy across all trials. (A) The effect of subsequent performance during the cue period, shown at an exemplary single sensor of interest, MLF51, whose middle left frontal location of the singe sensor is highlighted in red (right). (B) The effect of subsequent performance during the cue period averaged across all 270 MEG sensors. Box below highlights significant temporal cluster (∼583 ms) within the theta band (*p*<.001; format as in Figure 2). (C) The interaction of movement initiation and subsequent performance effects (i.e., the difference between the movement-initiation effects in accurate versus inaccurate trials), shown at an exemplary single sensor of interest, MLT15, whose posterior left middle temporal location is highlighted in red (right). (D) The interaction of movement initiation and subsequent performance effects averaged across all 270 MEG sensors. Box below highlights a significant temporal cluster (∼283 ms) within the theta band (*p*<.001; format as in Figure 2).

In a next step, we looked at subsequent memory effects in the passive pre-navigation planning or cue period (where participants were presented with a picture of a previously collected object) prior to active retrieval by comparing a median split of trials (within participants) corresponding to subsequently accurately versus inaccurately replaced objects. We had a strong a priori hypothesis of increased theta for performance, so we set our significance threshold at *p*<.001 uncorrected. Using a paired *t* test across participants, we found a significant subsequent memory-related theta power increase in the average signal of all sensors during the cue phase, peaked at 583 ms (*p*<.001 uncorrected, *t*(17) = 4.38). Induced theta oscillations were clearly visible for most of the epoch (Figure 3A–B). There was also a significant correlation (Pearson value: *p* = .027; *r* = −.519; Spearman value: *p* = .023; *r* = −.534; *df*(17)) between each participant's peak theta power from this contrast with their average distance error (Figure S4).

We also combined all movement onset and stationary epochs during the learning phase and ran a paired *t* test, dividing the trials based on the median split within participants according to subsequent performance on the object encountered during that trial. A significant theta effect was found for accurate versus inaccurate subsequent performance, peaked at 367 ms (*p*<.001 uncorrected, *t*(17) = 4.04). To distinguish whether the movement onset or stationary epochs contributed more to this effect, we tested for an interaction between movement onset or stationary epochs and subsequently well-performed versus poorly performed trials from the movement initiation analysis. The subsequent performance-related difference in theta power was greater for movement onset than stationary epochs (*p*<.001 uncorrected, peak at ∼283 ms; *t*(17) = 3.67; Figure 3C–D). No subsequent performance-related theta power increases were seen during stationary compared to movement onset epochs. Finally, there also appeared to be a significant increase in (∼9–12 Hz) alpha oscillatory power corresponding to movement onset in high-performing trials (Figure 3D). Recent work has shown theta and alpha oscillations in the hippocampus and perirhinal cortex are related to successful subsequent memory performance [30].

### fMRI Analyses

**Figure 4:**
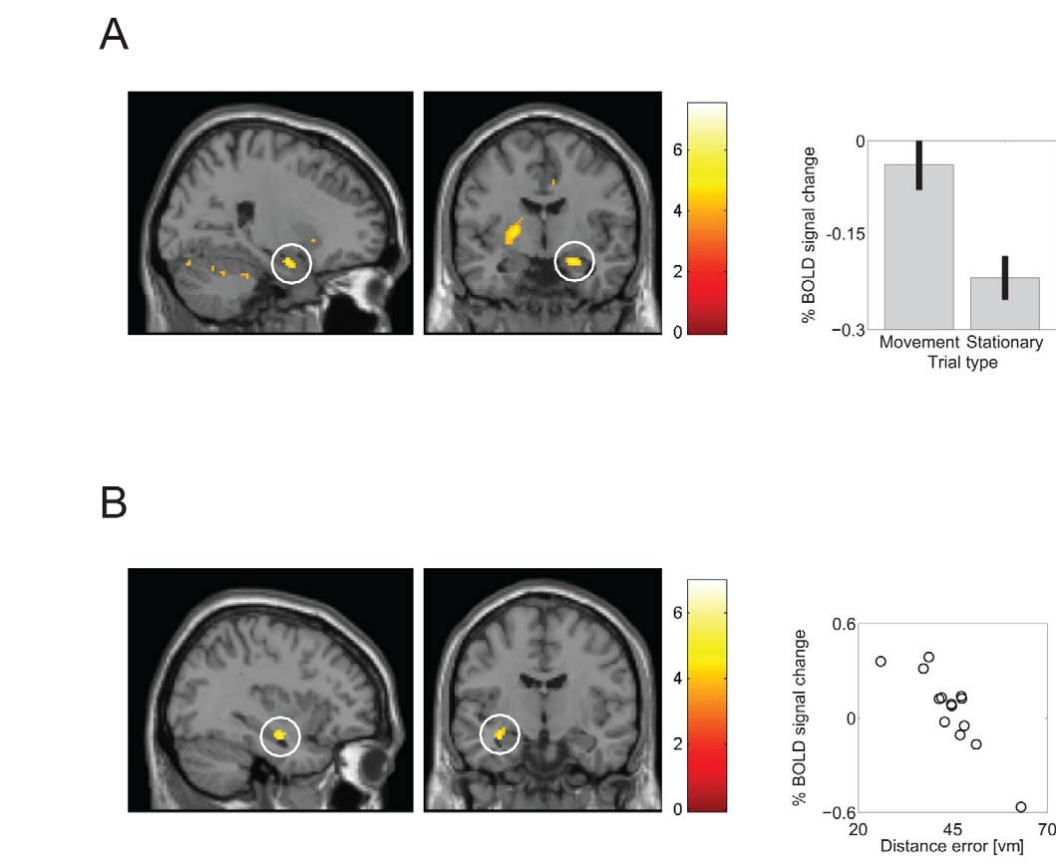
Movement- and performance-related fMRI effects. (A) Left: Sagittal and coronal slices showing right hippocampal activation for movement initiation compared to stationary periods (peak x = 24; y = −6; z = −18; Z-score = 3.83, thresholded at *p*<.001). Right: Percent signal change at right hippocampal peak-voxel averaged across 14 participants for movement and stationary periods (mean± SEM). (B) Left: Sagittal and coronal slices showing the correlation of left hippocampal activation during movement initiation with each participant's mean performance (peak voxel x = −32; y = −10; z = −14; Z-score = 3.66; thresholded at *p*<.001; right-hippocampal activation, not shown, has peak x = 40; y = −18; z = −14; Z-score = 3.20). Right: Percent signal change in left hippocampus for each participant plotted against his or her average replacement accuracy in virtual meters. Activations are overlaid on the SPM8 canonical single-participant T1 image.

Within our multi-modal neuroimaging approach, we ran follow-up fMRI analyses to corroborate our MEG results. We ran a one-sample *t* test on the contrast images for 1-s movement initiation periods versus stationary periods, at any point during virtual navigation within the two learning sessions, for the 14 participants who underwent fMRI scanning, using the uncorrected threshold of *p*<.001, *t*(13) = 3.85, for all contrasts. Using a whole brain univariate GLM we found the right hippocampus to be significantly more active for movement onset than stationary epochs (peak voxel: x = 24, y = −6, z = −18, Z-score = 3.83; see Figure 4A). We also observed activations in the bilateral cerebellum, inferior frontal gyrus, inferior parietal lobule, and basal ganglia (Table S1). In the reverse contrast, we saw increased bilateral posterior parahippocampal cortex activation for stationary periods compared to movement initiation (right peak: x = 18, y = −44, z = −10; Z-score = 4.38; left peak: x = −20, y = −54, z = −6, Z-score = 4.14). Thus, there is a transition from parahippocampal activation during stationary scene processing to hippocampal activation during movement initiation.

To follow up the movement-initiation finding, we correlated each participant's average subsequent accuracy (mean distance error) with the movement initiation fMRI effect (movement versus stationary contrast images). We found increased hippocampal activity associated with better performance (left peak: x = −32, y = −10, z = −14; Z-score = 3.66; right peak: x = 40, y = −18, z = −14; Z-score = 3.20, see Figure 4). Increased precuneus, bilateral inferior parietal lobule, ventral occipitotemporal area, and bilateral basal ganglia activity was also seen in this contrast (Table S2). No voxels showing increased activation for worse performance survived our threshold.

### Theta Source Analyses

**Figure 5:**
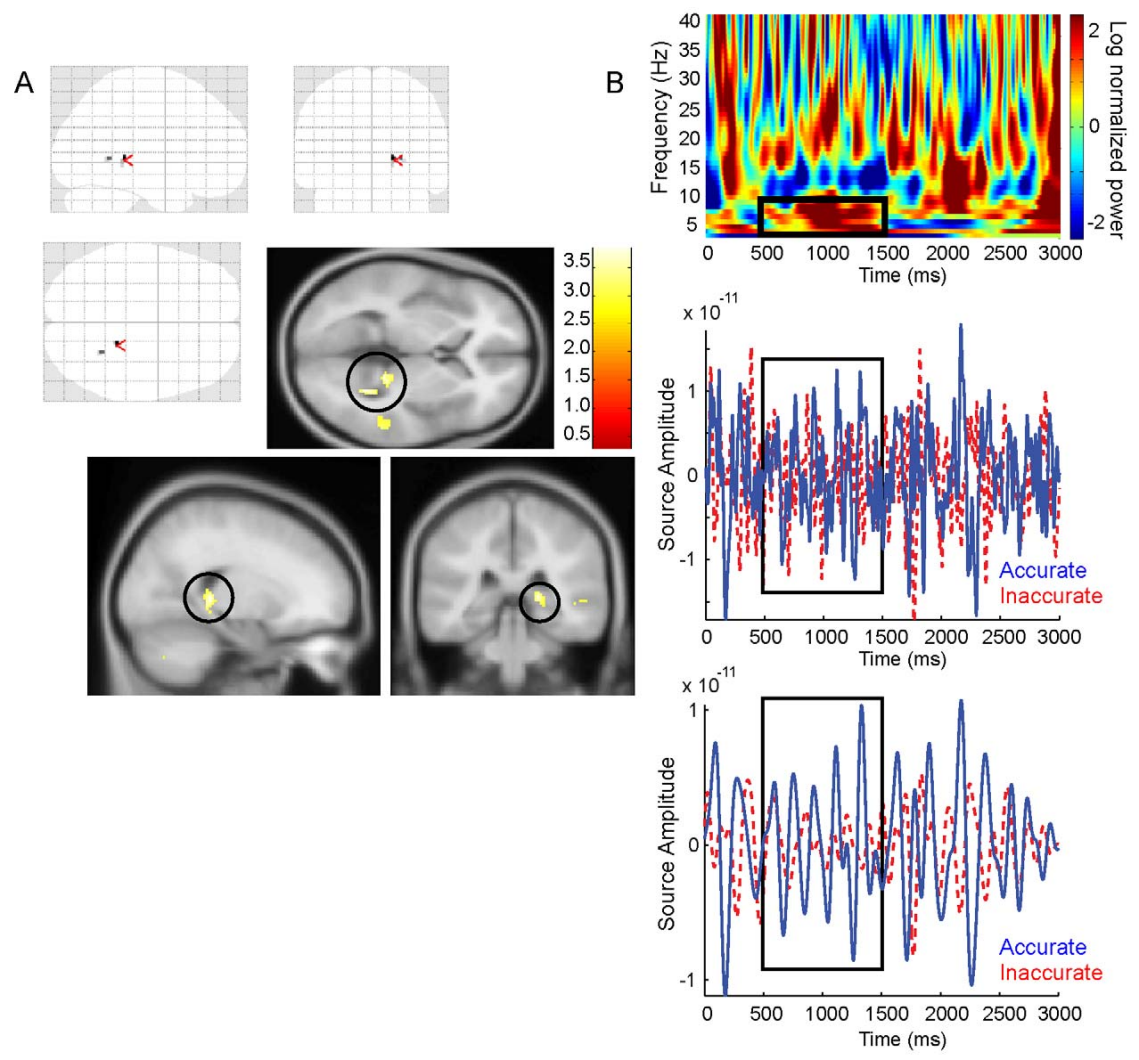
MEG source reconstruction of performance-related theta during the cue phase. (A) Top: “Glass brain” showing performance-related posterior right hippocampal source in the 4–8 Hz theta band from 500–1,500 ms within the 3-s Cue Period, which were the only significant sources of theta in the whole brain (peak-voxels: x = 18; y = −36; z = 4; Z-score = 3.26, highlighted by red arrows and x = 26, y = −50, z = 4; Z-score = 3.19, thresholded at *p*<.001, see Figure 3A–B for corresponding sensor-level plots). Bottom: Hippocampal sources shown overlaid on the SPM8 canonical average MNI 152 T1 image, significance threshold at *p*<.005 for display purposes. (B) Top: Single participant spectra from beamformer source extraction of a virtual sensor in right posterior hippocampus (x = 18; y = −36; z = 4). The black box shows the window used for the LCMV beamformer (500–1,500 ms; 4–8 Hz). Middle: Representative traces from the same participant and site for accurate trials (blue) and inaccurate trials (red). Bottom: Filtered versions of the traces (3.5–8 Hz). Source Amplitude and Power for these source extractions are measured in Ampere Meters.

We estimated anatomical sources for the 3-s Cue Period Subsequent Performance contrast image using a Linearly Constrained Minimum Variance (LCMV) beamformer algorithm [34] implemented in SPM8. We looked for theta (4–8 Hz) sources across the whole brain within the 500–1,500-ms theta effect time window (Figure 3B), at the uncorrected significance threshold of *p*<.001, *t*(16) = 3.686, for all contrasts. We found two significant peaks in the right posterior hippocampus (x = 18; y = −36; z = 4; Z-score = 3.26; x = 26; y = −50; z = 4; Z-score = 3.19, see Figure 5) and none elsewhere in the brain. We also conducted the same analysis on the 1-s Movement Initiation effects but saw no significant effects, possibly because of the transient (<500 ms) nature of the theta power change. We also used beamformer analyses to estimate the signal from the hippocampal coordinates of our fMRI movement initiation effect (MNI coordinates: x = 24, y = −6, z = −18) and a frontal midline region (MRI coordinates: x = 10; y = 30; z = 22). We observed strong theta increases (centered around ∼4 Hz) in the hippocampus and in the medial Prefrontal Cortex (centered around ∼6 Hz) during virtual movement initiation versus stationary trials (Figure S5).

## Discussion

We sought to investigate volitional movement-related theta oscillations in and their relation to self-directed memory encoding and hippocampal fMRI activity during an ecologically valid virtual spatial memory task using MEG. We found increased theta power during movement initiation (Figure 2A–B), an effect that was enhanced in familiar environments (Figure 2C–D). Additionally, we found performance-related theta power increases during navigation, which were stronger during volitional movement initiation than stationary periods (Figure 3C–D). We also observed theta power increases (Figure 3A–B) that were localized to the right hippocampus (Figure 5) during the static cue phase, where increases predicted subsequent spatial memory performance. fMRI functional localization of movement initiation periods revealed increased hippocampal activity (Figure 4A), and fMRI activations related to subsequent memory performance were also seen in the hippocampus during self-initiated movement (Figure 4B).

The increase in theta power at the initiation of volitional movement parallels the increased theta power seen in rodent hippocampus during the initiation of movement [1]–[2] and the dominant influence of motoric contributions versus cognitive factors in rodent studies of theta [8]. These findings corroborate previous findings of virtual movement-related theta oscillations in humans [18],[33],[35] and rodents [36]. In our task, the top movement speed was held constant, but there has been evidence that delta/theta power in the human hippocampus increases with virtual movement speed during navigation [21]. The increase in theta power in familiar environments compared to novel ones during the initiation of movement parallels the reduction in theta frequency (but not necessarily power) found when a rat enters a novel environment [6]. These changes were only found in response to environmental familiarity versus novelty, but not object familiarity versus novelty, supporting our hypothesis of theta power changes specifically in response to environmental novelty. This is consistent with the rodent literature where processing the novelty of the environment, rather than the objects within it, is specifically dependent on the hippocampus [37]–[39].

In addition to motoric and environmental factors, we also found links between theta power and cognitive performance, following previous studies in humans. Theta power in the (static) cue period (where participants coordinated the retrieval of object location prior to navigation) reflected subsequent replacement accuracy, consistent with previous MEG and EEG studies of human memory [13],[26]–[31]. The source of this theta effect was localized to the right hippocampus, in line with previous work [23],[40]–[43]. Notably, sensor-level peak theta power was higher in better performing participants, suggesting behavioural relevance.

We next examined the relationship between the movement-initiation-related theta during learning trials and subsequent performance in test trials. We found that the theta power difference between movement-initiation and stationary periods increased in trials in which there was more accurate subsequent memory performance.

Analysis of 1-s movement initiation periods with our fMRI functional localizer task demonstrated increased activity in the right hippocampus, in line with previous studies linking the right hippocampus to navigation [23],[40]–[43]. Our movement initiation fMRI contrast showed an overlap with a hippocampal-centered network including the cerebellum, lateral frontal areas, and IPL (inferior parietal lobule) concerned with the active encoding of item locations [11]. Notably, the main structures active during our movement initiation fMRI localizer, the basal ganglia and the cerebellum, show theta oscillatory synchronization to the hippocampus during learning in small mammals [44]–[46], and functional connectivity has been observed between hippocampus and cerebellum [11],[47]–[48] and basal ganglia [49] in human fMRI. Interestingly there was a notable dissociation of navigation-related fMRI activations in the medial temporal lobe: in contrast to the hippocampal activation, which was specific to movement initiation, the reverse fMRI contrast (stationary versus movement initiation) showed bilateral activation of the posterior parahippocampal cortex, consistent with its role in static scene processing [50].

Increased bilateral hippocampal fMRI activity related to movement initiation was seen in participants who showed better subsequent memory for the object locations. This finding suggests a link between the process of self-directed movement initiation and encoding efficiency within this task, consistent with previous findings relating hippocampal activation during encoding with subsequent memory performance ([51]–[52]; for review see [53]).

The presence of a performance-related increase in theta power during active exploration provides a possible link to human behavioural and fMRI studies of active versus passive learning enhancements derived from the hippocampal-dependent volitional control network [11]. Voss and colleagues demonstrated that the hippocampus supports volitional control of exploratory behaviour so as to optimize learning of object locations. There is significant overlap between this concept and the hypothesized functional role for hippocampal theta in behavioural control of exploration, active movement during a spatial memory task being an example of the active control of learning [2],[54]–[55]. Thus in accordance with O'Keefe and Nadel, we propose that movement-related theta may aid in signalling the potential for volitional control of encoding, which is consistent with findings in rodents showing increased hippocampal theta power for volitional versus deterministic movement [56] and humans showing increased hippocampal theta power for goal-directed versus aimless movements [20]. Notably, the possibility that movement-related theta interacts with performance-related influences on theta is further supported by our finding that both theta power and hippocampal activity for movement initiation relative to stillness correlate with performance during navigation, a time period where the participant has active control over how he or she encodes a particular spatial representation.

Although we measured theta power and hippocampal blood oxygen level-dependent (BOLD) signal increases in the same 1-s time periods corresponding to navigation behaviour and memory performance in the same participants, we do not assume a direct correlation between BOLD and theta oscillatory power. The relationship between BOLD and hippocampal theta is unclear. Past work by Ekstrom and colleagues has shown decoupling between hippocampal BOLD and theta, despite findings that BOLD and theta may negatively correlate elsewhere in the brain [57]–[59]. Additionally, it is important to emphasize that even the MEG signals on temporal sensors were not being measured specifically from the hippocampus, although we note that the analogous aspects of theta oscillations in rodents [1]–[2],[6],[60] are known to depend on the septo-hippocampal system. Unlike the performance effect during the 3-s Cue Period, we were not able to localize the source of the movement-initiation related theta increase, possibly because its transient nature did not allow good frequency resolution. In our experiment, the participants typically moved in 1-s periods, precluding the investigation of longer time windows. Longer periods of continuous virtual movement would allow us to better investigate induced low frequency activity in the hippocampus, as seen in other experiments [18],[21].

Given the literature in rodents [61]–[62] and humans [63] showing hippocampal-prefrontal interactions during spatial navigation, we investigated the MEG signal from both regions. Consistent with this literature, we found virtual movement-related theta in both the frontal midline and hippocampus (Figure S5). Numerous studies have also found task-related midline frontal theta power and phase changes during successful memory encoding and maintenance (for reviews see [64]–[66]). These performance effects are thought to underpin key neocortical-hippocampal interactions in learning and memory. Future research will investigate the probable midline frontal and hippocampal sources of the theta-band signals reported here, and their potential interactions with other brain regions [4],[66]–[68].

Our performance correlates of theta power complement previous MEG work looking at medial temporal lobe theta power before the onset of an encoding trial [28]–[30] and increased theta power for successfully encoded memories during mnemonic processing [13]–[14],[26]–[27],[31]. Furthermore, by showing enhanced subsequent memory effects during virtual movement over stationary periods, this is the first study to our knowledge that implicates self-initiated movement-related theta increases with self-directed learning.

The hippocampus and corresponding theta oscillations have been hypothesized as a network hub [69]–[70] and global signal integrator [55] for information from around the brain. The potential role for hippocampal theta for guiding self-directed learning paves the way to investigate how theta and hippocampal-related active control mechanisms interact with a wide range of networks responsible for dynamic evaluative behaviours like planning and novelty processing [31],[71]–[72] in which theta power and synchrony changes have been observed. For instance, evaluative behaviours relating to emotion and anxiety have been associated to theta [73]. Theta synchronization in rodents has been observed between the hippocampus and the amygdala during fear learning [74] and between the hippocampus and medial prefrontal cortex during anxiety [75]. Hippocampal theta dysfunction related to learning control dynamics during encoding could possibly represent core pathology in mental illnesses, where feelings of helplessness (i.e., lack of control) in certain environments are common, such as post-traumatic stress disorder (PTSD) and depression [76]–[77].

In summary, our results indicate the key role movement and the resulting self-initiated dynamic control of spatial encoding have in generating human theta oscillations and supporting hippocampal mnemonic function. Further studies into oscillatory characteristics and functional networks associated with the hippocampus and volitional learning will be necessary to clarify the role hippocampal theta has in the control of active learning. By using an interactive ecologically realistic experimental task and multi-modal neuroimaging to investigate hippocampal function, our results show that an analogue of Type I theta in the rodent hippocampus can be found in humans and likely serve to coordinate self-directed learning.

## Materials and Methods

### Participants

Twenty right-handed male participants (mean age = 23.5 years, SD = 5.06, range 18–35) gave written consent and were compensated for performing the experimental task, as approved by the local Research Ethics Committee. All participants were right-handed with normal or corrected-to-normal vision and reported to be in good health with no prior history of neurological disease. One participant was excluded from the analysis because of equipment malfunction, and another was excluded from analyses because of signal artifacts. Eighteen right-handed male participants were therefore analyzed in the MEG dataset, with one being excluded from the source reconstruction because of a problem with co-registration between the MEG head position and structural MR image. Fourteen of these also participated and were analyzed for the fMRI functional localizer.

### Virtual Reality Environment

UnrealEngine2 Runtime software (Epic Games) was used to present a first-person perspective viewpoint of four different environments, a dry sandy environment surrounded by dunes (practice environment), a snowy grassy urban environment surrounded by skyscrapers, a rocky desert environment surrounded by pyramids, and a grassy plane surrounded by a circular cliff with a background of mountains, clouds, and the sun. In all environments, background cues were projected at infinity to provide orientation but not location within the arena. Participants moved the viewpoint by using their right hand to press keys to move forward or turn left or right. The viewpoint is ∼2 virtual meters above the ground, and all four environments had the same arena size (area). Virtual heading and locations were recorded every 25 ms.

### Stimuli, Task, and Trial Structure

The experiment was composed of eight sessions. The first two sessions were practice sessions using the same virtual desert environment, conducted on a laptop outside the scanner. The participants first familiarized themselves with the environment by navigating around and then collecting objects in the environment by running them over and then being tested on their previous location [23]. These practice sessions lasted for about 2–3 min.

In the MEG scanner, an individual trial consisted of a participant being randomly placed in an environment and having to navigate towards an object to collect and remember its location (average duration ∼10.6 s). Participants had three trials to learn the location for each of the six objects. Next, the participants were presented with a grey screen that read “Please Blink” for a 1.5-s blink phase, then a 1-s intertrial baseline where a crosshair was presented on a grey screen. During the learning period of the session, there were 18 trials each consisting of a learning phase blink phase, and baseline intertrial interval (Figure 1). After the learning period of a session was completed, there was a 30-s inter-phase rest period, when instructions on the next phase (test phase) were presented. The test phase for the location of each of the six collected objects started with a 3-s period in which an object was presented on a grey background (cue phase) (Figure 1). Participants were then randomly placed in the environment and told to navigate to the spot where they believed the pictured object had been located (average duration ∼15.1 s). They then pressed a button to “drop” the object or indicate its previous location. Once the button was pressed a grey screen appeared that read “Please Blink” for a 1.5-s blink phase, followed by a 1-s intertrial baseline.

In the fMRI functional localizer component, participants had functional scans during performance of two different learning period sessions. After they had completed both functional localizer sessions, participants had separate test sessions (i.e., first test for first learning session, second test for second learning session) for each respective learning session to gauge subsequent performance, where they were not scanned. The functional localizer had the same trial structure and amounts as the MEG experiment with the exception of an extended 4-s inter-trial interval (without a blink phase) to account for the timescale of the BOLD signal.

### Details of Procedure and Design

Participants were instructed that they were going to navigate through a virtual environment over multiple sessions using a button box, and that they would have to pick up several different objects (six) in the environment, three times each (three objects, three times each for the two practice sessions). The order of trials was randomized but (unknown to participants) separated into three mini-blocks [23]. Object location never changed within a session. After they completed this exploration phase, they were tested at the end in a test period by having to navigate to where they thought the object had been located and press a button.

During MEG scanning, a new environment was presented and then re-presented at the next session as a familiar environment. This occurred on four occasions (three within the MEG), so that half of the eight environments in the experiment were novel and the others were familiar (Figure 1). The order of environments in the MEG sessions was randomized across participants. Each environment arena had the same distance area, but did have its own unique shape (square, circle, triangle, and rectangle) to differentiate the environment. It is also important to note that virtual movement during navigation trials consisted of continued forward button pressing causing a constant speed of forward motion.

As a control measure for environmental novelty independent of other novelty effects (i.e., object-novelty) during movement initiation, participants were presented with counterbalanced familiar or novel objects within each environment. Following the practice sessions, the objects presented in an environment were comprised of objects that the participants had either collected (“familiar”) or not collected (“novel”) in a previous session. Familiar objects were first introduced during the practice session outside of the scanner.

For the fMRI functional localizer, there was no manipulation of environmental or object novelty. The two sessions were conducted in the circle mountain environment used in the MEG experiment with all novel objects for both sessions. Otherwise, the procedure was analogous to the MEG design.

### MEG Acquisition

Recordings were made in a magnetically shielded room with a 275-channel Canadian Thin Films (CTF) system with superconducting quantum interference device (SQUID)-based axial gradiometers (VSM MedTech Ltd.) and second-order gradients. Neuromagnetic signals were digitized continuously at a sampling rate of 480 Hz and behavioural responses were made via an MEG-compatible response pad. We used a high pass filter of 0.1 Hz and a low pass filter of 120 Hz. Head positioning coils were attached to nasion, left, and right auricular sites to provide anatomical coregistration. Coils were energized before and after each session to determine head movement and position within the MEG dewar. At acquisition for some participants, some sensors were corrupted. As a result, only 270 of the 275 sensors were analyzed to keep data consistent across all participants.

### MEG Data Analysis

Data were analyzed with SPM8 (Wellcome Trust Centre for Neuroimaging, London) [32] and FieldTrip toolbox (Donders Centre for Cognitive Neuroimaging, Nijmegen, the Netherlands) [78] within MATLAB 7 (The MathWorks).

### Pre-Processing

Although the total trial duration varied, to assess the effects of virtual movement and novelty, we defined fixed-length segments where the participant's state was comparable across trials at any period (learning and test) in the experiment. Epochs corresponding to movement initiation were defined as −200 to 800 ms relative to the initiation of forward displacement at any point in any navigation (learning and test periods) trial, where participants moved for at least 1,000 ms. As an equal length comparison condition within the experiment, stationary epochs were characterized as 500 ms after a participant had stopped moving for a duration of 1,000 ms without any forward displacement at any point during any navigation (learning and test periods) trial. Both of these windows were extended by an additional 1,000 ms on either side for analysis purposes. Importantly, participants' top speed remained constant during all movement periods. Within the movement-based analyses, epochs were defined as belonging to one of ten different conditions in which movement and stationary epochs were separated by whether they occurred within novel environments or during novel object trials: movement onset during a familiar object trial, movement onset during a novel object learning trial, stationary period during a familiar object learning trial, stationary period during a novel object learning trial, and the 1,000 ms inter-trial baseline condition. To look at environmental novelty, these five conditions were also defined the same way, but the extra factor of session environment (familiar or novel) was added.

For the subsequent memory analysis during exploration (1-s movement and stationary epochs), the epochs were divided into well-performed trials and poorly performed trials at a median split for each participant. In this analysis there were five trial types: movement onset during a well-performed trial, stationary period during a well-performed trial, movement onset during a poorly performed trial, stationary period during a poorly performed trial, and the inter-trial baseline.

On average each participant had 178.2 movement onset epochs versus 197.8 stationary epochs with 138 baseline epochs. For the environmental novelty contrast, each participant had on average 90.7 virtual movement initiation epochs in a novel environment, 87.5 virtual movement onset epochs in a familiar environment, 105.1 stationary epochs in a novel environment, 92.7 stationary epochs in a familiar environment, 69 baseline epochs in a familiar environment, and 69 baseline epochs in a novel environment. For object novelty, each participant had an average of 85.2 virtual movement onset epochs measured during novel object trials, 93 virtual movement onset epochs measured during familiar object learning trials, 98.3 stationary trials measured during novel object trials, and 99.5 stationary trials measure during familiar object trials. For the subsequent memory analysis during navigation (an average total of 273.1 epochs per participant), there were 62.9 virtual movement onset epochs during subsequent accurately performed learning trials, 62.9 virtual movement onset epochs during subsequent inaccurately performed learning trials, 73.3 stationary epochs during subsequent accurately performed learning trials, 73.9 stationary inaccurately performed learning trials, and 102 baseline trials.

For the cue period analysis, the 36 3-s-long cue periods were divided into well-performed trials versus poorly performed trials. There was also a 1-s baseline prior to each 3-s-long cue period trial.

### Time-Frequency Analysis

Two seconds of padding (one second at each end) were added to the movement epochs to capture more theta cycles. Additionally, trials were inspected and removed if they contained eyeblink artifacts. Data were downsampled to 120 Hz, and a five-cycle morlet wavelet time-frequency analysis ranging from 3 to 48 Hz with a frequency resolution of 1 Hz was conducted. Lower delta frequencies (below 3 Hz) were not measured because of the limited number of possible cycles in the short trial length and edge effects. The same analysis stream was followed for the cue phase analysis, with the exception that cue epochs lasted 3 s with another 1 s pre-stimulus baseline, in which the participant was intended to stare at a fixation cross. With 3-s-long epochs there was a lower chance of edge effects in the delta frequency band (1–4 Hz), so we extended our time-frequency analysis to 2 to 48 Hz. Still, to avoid edge effects, the 3-s-long time window was reduced to 2.5 s.

Next, a weighted average (i.e., making sure that trial numbers between participants were weighted into calculated averages) of time-frequency trials was calculated within participants and session by condition. Data were then log transformed and baseline corrected (i.e., the data were expressed as a multiple of baseline power). For the movement analyses, the baseline was computed from a set of 1,000 ms baseline trials. For the cue period analysis the first 1,000 ms prior to the cue onset were used as a baseline.

Time-frequency data were then converted into Neuroimaging Informatics Technology Initiative (NIfTI) format. This produced a 3-D image of Channel Space×Time. The frequency dimension was averaged across the theta frequency band (4–8 Hz) based on our a priori hypotheses.

### MEG Statistical Analysis

For the virtual movement effect, a paired *t* test of virtual movement onset and offset conditions was conducted with a Family Wise Error (FWE) corrected cluster threshold of *p*<.05, because of our previous event-related hypothesis to the effect and time scale of theta during movement initiation [32]. For object and environmental novelty movement effects, a one-way ANOVA for the 2×2 factors of object novelty versus environmental novelty was used. The same statistical threshold as for the virtual movement effect was also used. The (cue phase and subsequent memory) performance effects were calculated with a paired *t* test with a threshold of *p*<.001 uncorrected without the cluster correction because of our strong a priori hypothesis from the past literature looking at theta and memory performance and the lack of a specific event-related hypothesis for the time-scale of power changes [17].

### MEG Source Reconstruction

The linearly constrained minimum variance scalar beamformer spatial filter algorithm (34) from SPM8 was used to generate source activity maps in a 10 mm grid. Coregistration to the MNI coordinates was based on three fiducial points: nasion and left and right preauricular. The forward model was derived from a single-shell model [79] fit to inner skull surface of the inverse normalized SPM template. The beamformer source reconstruction is based on two stages [80]. First, based on the data covariance and lead field structure, weights are calculated which linearly map sensor data to each source location. Second, a summary statistic based on the change in source power or amplitude over experimental conditions is calculated at each voxel. In this case the summary statistic at each voxel is the change in source power in the 4–8 Hz band normalized by the projected sensor white noise power. In this case, the periods under comparison were accurately and inaccurately remembered trials in a time window 500–1,500 ms after Cue onset (i.e., within the cue period). For each participant these summary statistic images were entered into a second-level one-sample *t* test in SPM8. A statistical threshold of *p*<.001 was used.

The beamformer source extraction for the movement initiation effect was measured from two locations (medial Prefrontal Cortex, x = 10; y = 30; z = 22; right anterior Hippocampus, x = 24; y = −6; z = −18;) and projected through a spatial filter constructed from the covariance matrix comprising 1-s Navigation conditions with 1-s of padding on either side for three conditions: movement, stillness, and pre-navigation baseline. Subsequently, the same time-frequency wavelet analysis from the sensor-level analyses was run on these two virtual sensor locations from the mPFC and hippocampus.

### fMRI Acquisition

Functional images were acquired on a 3T Siemens Allegra scanner. Blood oxygenation level dependent (BOLD) T2*-weighted functional images were acquired using a gradient-echo EPI pulse sequence acquired obliquely at −45 degrees with the following parameters: repetition time, 2,880 ms; echo time, 30 ms; flip angle, 90 degrees; slice thickness, 2 mm; interslice gap, 1 mm; in-plane resolution, 3×3 mm; field of view, 64×72 mm^2^; 48 slices per volume. A field-map using a double echo FLASH sequence was recorded for distortion correction of the acquired EPI [81]. After the functional scans, a T1-weighted 3-D MDEFT structural image (1 mm^3^ resolution) was acquired to co-register and display the functional data.

### fMRI Preprocessing

All preprocessing and analyses were performed with SPM8 (www.fil.ion.ucl.ac.uk/spm). All individual structural images underwent segmentation (into grey matter, white matter, and cerebro-spinal fluid), bias correction, and spatial normalization to the MNI template using “unified segmentation” [82]. Using the Montreal Neurological Institute (MNI) template brain, the first six EPI volumes were discarded to allow for T1 equilibration. EPI images had distortion correction and were realigned spatially to the time series' first image based on the collected field map [83] and the interaction of motion and distortion using the Unwarp routines in SPM [82],[84]. Functional images were normalized based on the spatial parameters derived from the normalization of their structural images. Normalized EPI images were spatially smoothed with an 8 mm isotropic FWHM Gaussian kernel. Data were high pass filtered at 128 s. All coordinates are in MNI space.

### fMRI Data Analysis

Statistical analyses were performed using a univariate general linear model (GLM) with a rapid event-related experimental design. There were two 1-s conditions of interest that were the same as the MEG, movement onset (initiation) and movement offset (no movement) conditions, which were modelled as a boxcar function (duration of 1 s) and convolved with the canonical hemodynamic response function (HRF) to create regressors of interest. Participant-specific beta values (parameter estimates) were calculated for each voxel, and the respective contrast images (movement onset versus offset) were entered into one-sample *t* tests in a second-level random-effects analysis. In a second analysis across participants, the movement onset versus offset contrast images were correlated with each participants' mean object replacement performance (mean distance error in virtual meters) during the test phase. Based on our strong a priori hypothesis about the hippocampus, we chose the threshold of *p*<.001 (uncorrected for multiple comparisons) with an extent threshold of 5 voxels.

## Supporting Information

Figure S1 Related to Figure 1. Top: Object replacement performance (distance error) performance for novel versus familiar objects. Bars show standard deviation. Bottom: The negative trend for object replacement across MEG sessions. Bars show standard error.
(TIF)

Figure S2 Related to Figure 1. A flow chart of the MEG time-frequency data analysis stream.
(TIF)

Figure S3 Related to Figure 2. Top: Single participant movement initiation spectral theta effect from same single Sensor (MRT16) shown in Figure 2A. Middle: Representative individual traces from sensor MRT16 showing the difference in the same time window between movement (blue) and still (red) periods in the same single subject. Bottom: Filtered (3–10 Hz) individual trace difference in theta oscillatory activity during movement initiation (blue) and stillness (red). Field intensity for all traces in Tesla.
(TIF)

Figure S4 Related to Figure 3B. Correlation between peak average cue performance-related theta power for each participant and his or her overall distance error across 18 participants. *p* = .027, *r* = −.519, *df*(17), Spearman correlation: *p* = .023, *r* = −.534.
(TIF)

Figure S5 Related to Figure 2. Beamformer leadfield source extraction from Right Hippocampus (fMRI coordinates from Figure 4A; x = 24; y = −6; z = −18) and medial PFC (x = 10; y = 30; z = 22) to compare midline prefrontal and hippocampal theta during the Movement Initiation effect shown on the sensor level in Figure 2A–B.
(TIF)

Table S1 Related to Figure 4A. Significant fMRI activations for the movement initiation contrast, at the p<.001 uncorrected threshold.
(DOC)

Table S2 Related to Figure 4B. Significant fMRI activations for the movement initiation contrast correlated with each participant's replacement performance (overall distance error), at the *p*<.001 uncorrected threshold.
(DOC)

## Abbreviations

BOLD: blood oxygen level-dependent
CTF: Canadian Thin Films
fMRI: Functional Magnetic Resonance Imaging
FWE: Family Wise Error
GLM: general linear model
HRF: hemodynamic response function
LCMV: Linearly Constrained Minimum Variance
LFP: local field potential
MEG: Magnetoencephalography
MNI: Montreal Neurological Institute
NIfTI: Neuroimaging Informatics Technology Initiative
PTSD: post-traumatic stress disorder
SQUID: superconducting quantum interference device
