# Supplementary figures and images for: Movement-Related Theta Rhythm in Humans: Coordinating Self-Directed Hippocampal Learning
Source: PLoS Biol. 2012 Feb 28;10(2):e1001267. doi: 10.1371/journal.pbio.1001267 (PMC3289589; doi:10.1371/journal.pbio.1001267)

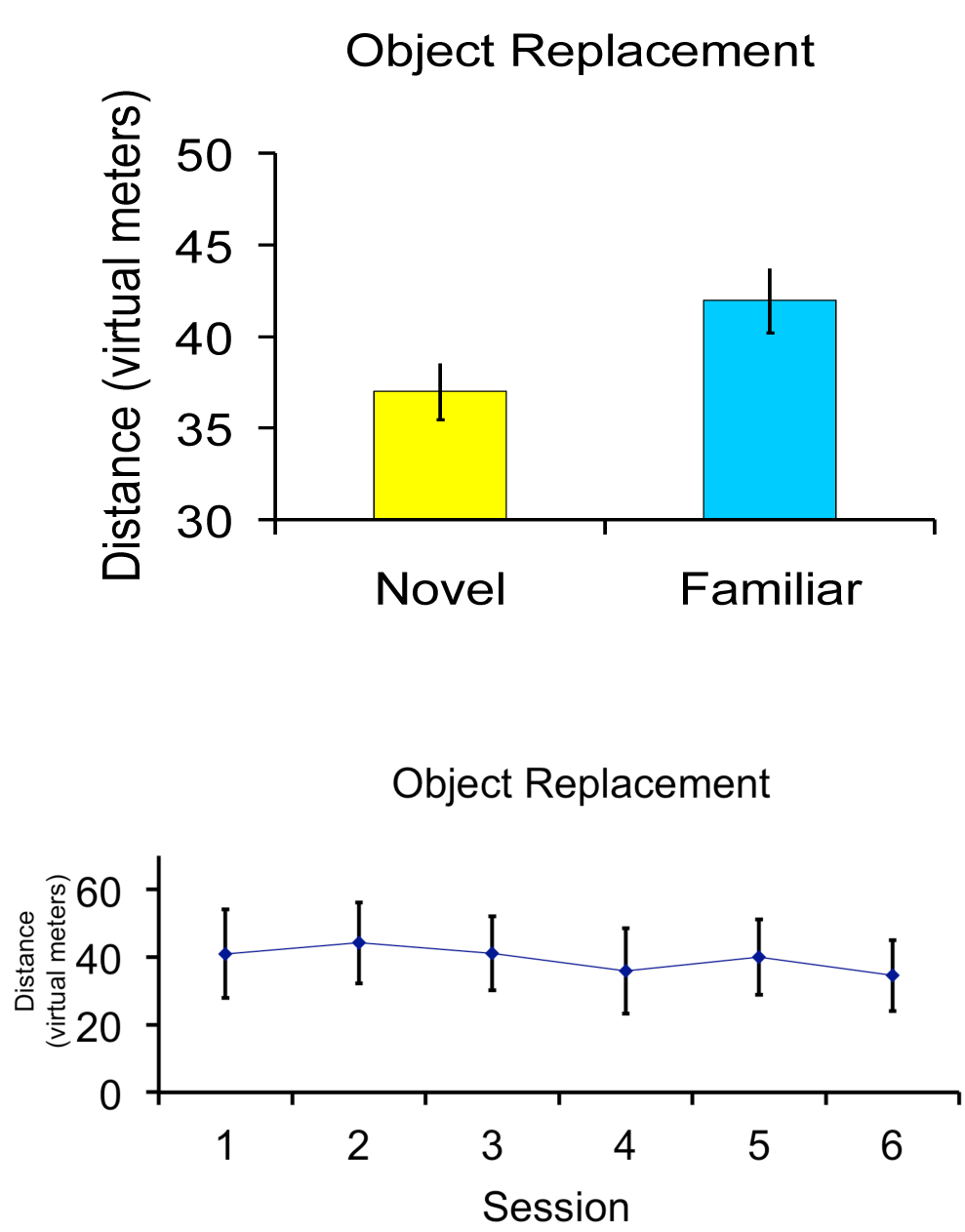

Supplement: Figure S1 — Related to Figure 1. Top: Object replacement performance (distance error) performance for novel versus familiar objects. Bars show standard deviation. Bottom: The negative trend for object replacement across MEG sessions. Bars show standard error. (TIF) [file pbio.1001267.s001.tif]

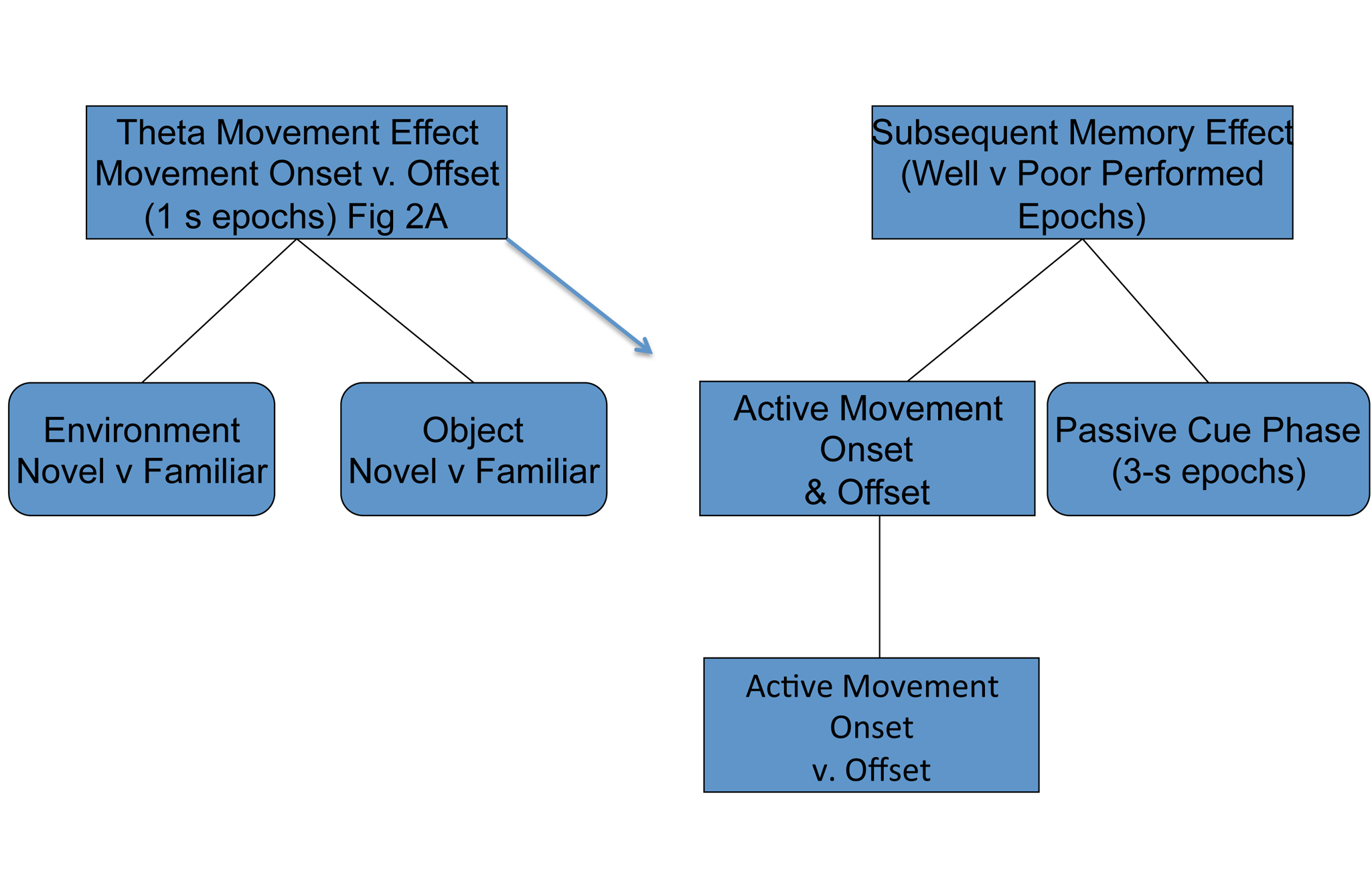

Supplement: Figure S2 — Related to Figure 1. A flow chart of the MEG time-frequency data analysis stream. (TIF) [file pbio.1001267.s002.tif]

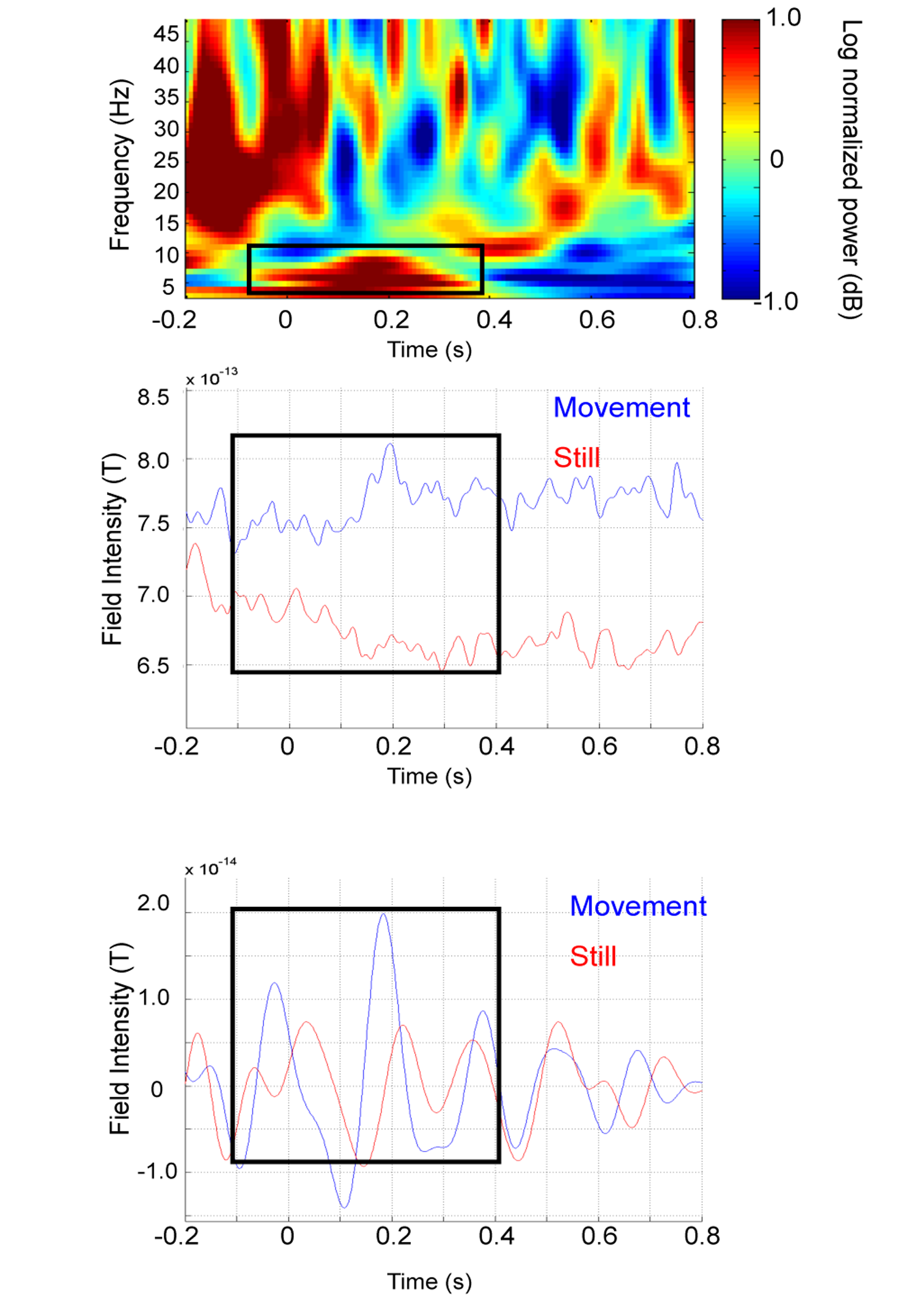

Supplement: Figure S3 — Related to Figure 2. Top: Single participant movement initiation spectral theta effect from same single Sensor (MRT16) shown in Figure 2A. Middle: Representative individual traces from sensor MRT16 showing the difference in the same time window between movement (blue) and still (red) periods in the same single subject. Bottom: Filtered (3–10 Hz) individual trace difference in theta oscillatory activity during movement initiation (blue) and stillness (red). Field intensity for all traces in Tesla. (TIF) [file pbio.1001267.s003.tif]

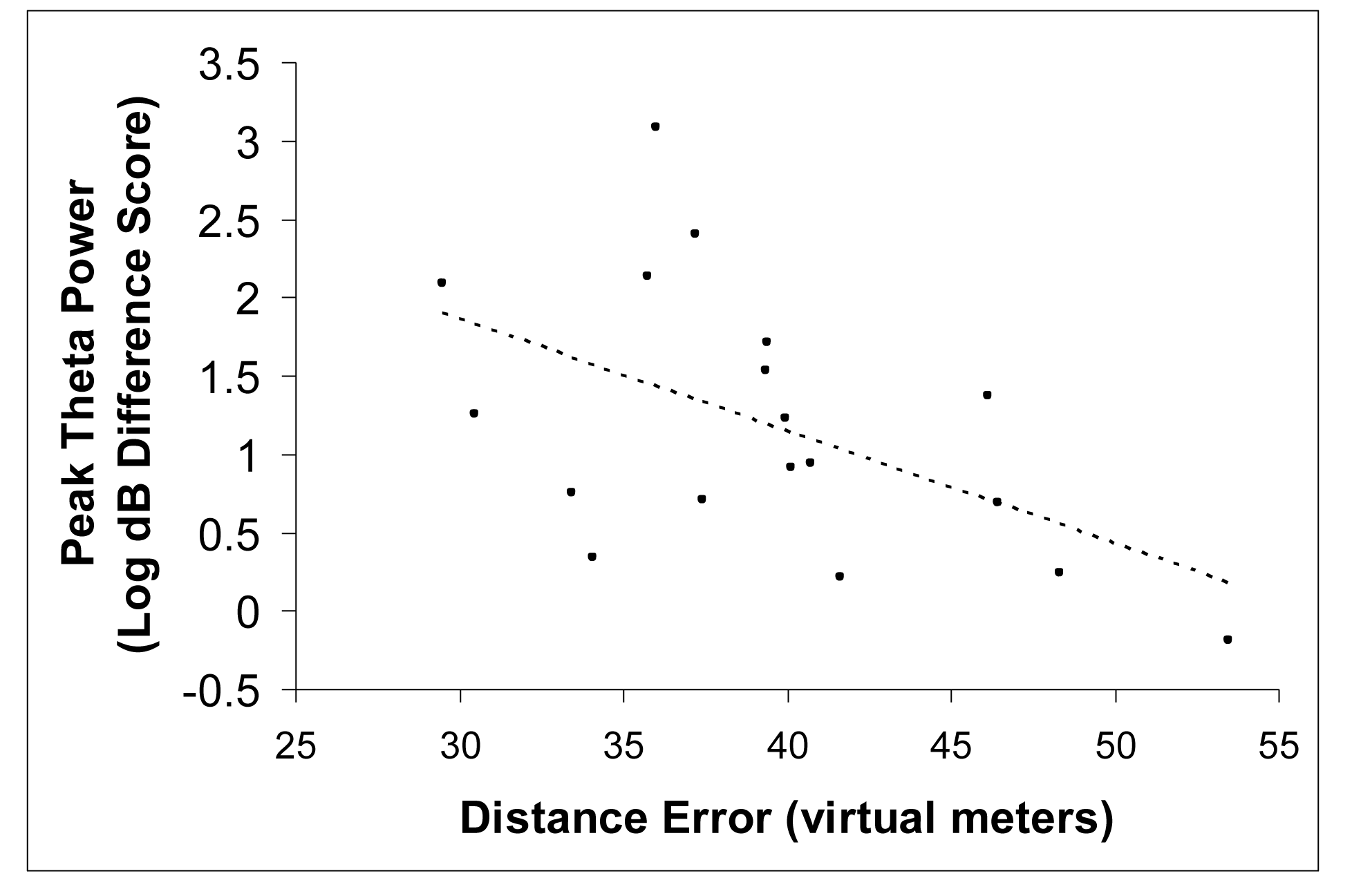

Supplement: Figure S4 — Related to Figure 3B. Correlation between peak average cue performance-related theta power for each participant and his or her overall distance error across 18 participants. p = .027, r = −.519, df(17), Spearman correlation: p = .023, r = −.534. (TIF) [file pbio.1001267.s004.tif]

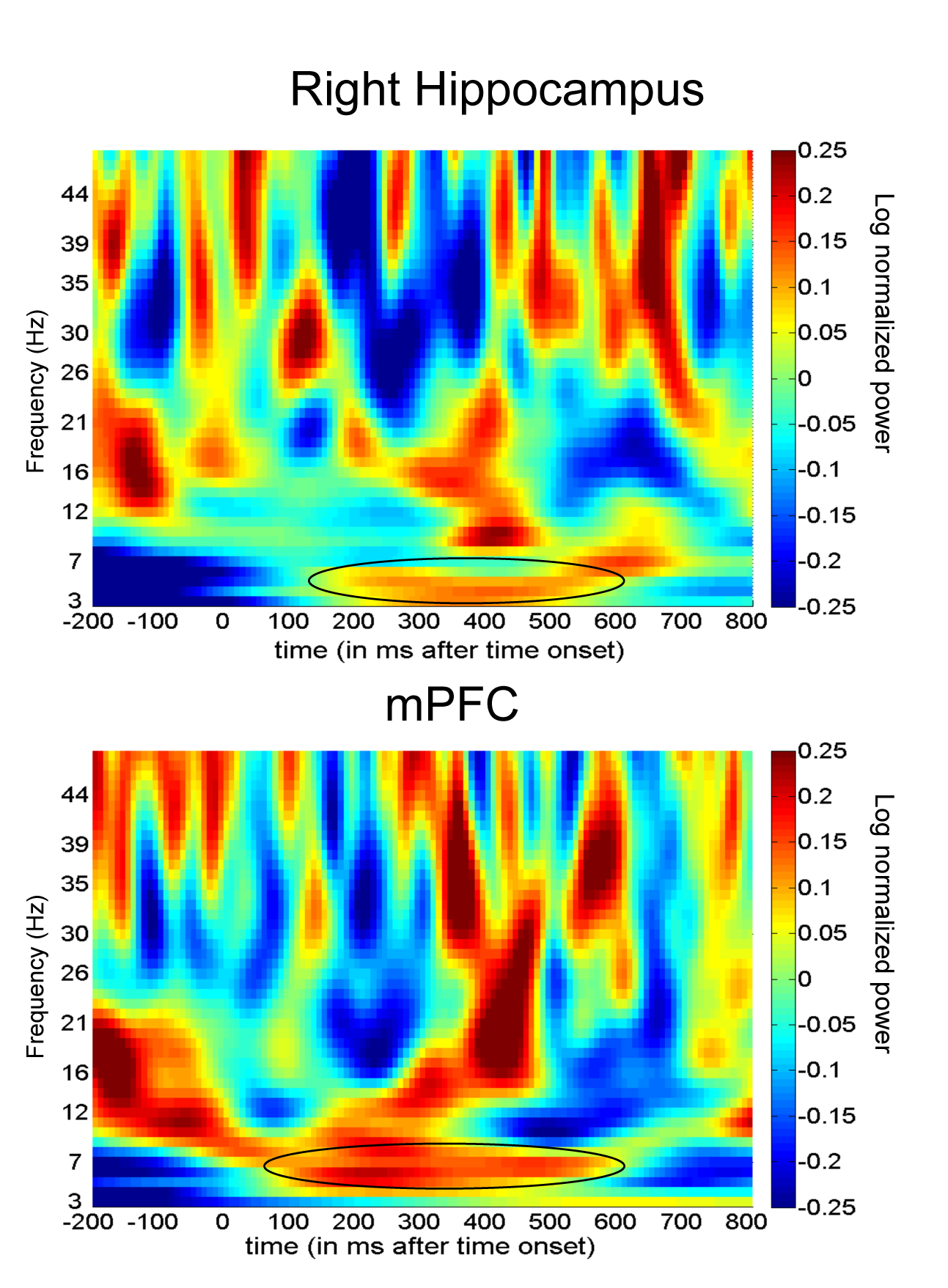

Supplement: Figure S5 — Related to Figure 2. Beamformer leadfield source extraction from Right Hippocampus (fMRI coordinates from Figure 4A; x = 24; y = −6; z = −18) and medial PFC (x = 10; y = 30; z = 22) to compare midline prefrontal and hippocampal theta during the Movement Initiation effect shown on the sensor level in Figure 2A–B. (TIF) [file pbio.1001267.s005.tif]
