# Supplementary material for: Movement-Related Theta Rhythm in Humans: Coordinating Self-Directed Hippocampal Learning
Source: PLoS Biol. 2012 Feb 28;10(2):e1001267. doi: 10.1371/journal.pbio.1001267 (PMC3289589; doi:10.1371/journal.pbio.1001267)
Supplement: Table S1 — Related to Figure 4A. Significant fMRI activations for the movement initiation contrast, at the p<.001 uncorrected threshold. (DOC) [file pbio.1001267.s006.doc]

Table S1. Movement Initiation fMRI Effect

| **Region** | ***x*** | ***y*** | ***z*** | **Z-score** |
| --- | --- | --- | --- | --- |
| L Cerebellum | -34 | -48 | -32 | 4.59 |
| R Cerebellum | 30 | -40 | -28 | 3.95 |
| R Superior Parietal Lobule | 16 | -78 | 48 | 4.49 |
| L Striatum | -22 | -8 | 6 | 4.06 |
| R Striatum | 26 | 14 | -2 | 3.58 |
| Cingulate Gyrus | -4 | -22 | 42 | 3.98 |
| R Inferior Frontal Gyrus | 56 | 26 | 2 | 3.97 |
| L Inferior Frontal Gyrus | -60 | 8 | 26 | 3.33 |
| R Hippocampus | 24 | -6 | -18 | 3.83 |
| R Insula | 44 | 12 | -8 | 3.76 |
| Midbrain | 10 | -24 | -26 | 3.76 |
| R Lateral Occipital Area | 36 | -84 | -6 | 3.64 |
| L Lateral Occipital Area | -48 | -62 | -2 | 3.60 |
| R Caudate | 16 | 4 | 14 | 3.30 |
| L Inferior Parietal Lobule | -30 | -46 | 64 | 3.21 |
