# Supplementary material for: Movement-Related Theta Rhythm in Humans: Coordinating Self-Directed Hippocampal Learning
Source: PLoS Biol. 2012 Feb 28;10(2):e1001267. doi: 10.1371/journal.pbio.1001267 (PMC3289589; doi:10.1371/journal.pbio.1001267)
Supplement: Table S2 — Related to Figure 4B. Significant fMRI activations for the movement initiation contrast correlated with each participant's replacement performance (overall distance error), at the p<.001 uncorrected threshold. (DOC) [file pbio.1001267.s007.doc]

Table S2. Movement and Performance fMRI Effect

| **Region** | ***x*** | ***y*** | ***z*** | **Z-score** |
| --- | --- | --- | --- | --- |
| Retrosplenial Cortex | -6 | -44 | 0 | 4.33 |
| R Ventral Occipitotemporal Cortex | 56 | -54 | -4 | 3.93 |
| R Inferior Parietal Lobule | 66 | -34 | 20 | 3.92 |
| L Inferior Parietal Lobule | -56 | -68 | 8 | 3.60 |
| L Hippocampus | -34 | -10 | -16 | 3.66 |
| R Hippocampus | 40 | -18 | -12 | 3.20 |
| L Globus Pallidus | -8 | 10 | -6 | 3.49 |
| R Middle Temporal Gyrus | 56 | -16 | -16 | 3.48 |
| L Middle Temporal Gyrus | -42 | -66 | 6 | 3.47 |
| R Caudate | 12 | 8 | 2 | 3.37 |
| L Secondary Visual Cortex | -12 | -86 | 24 | 3.22 |
